# Retrospective comparison of two distinct dual-induction strategies in pediatric kidney transplantation: three doses vs. a single dose of rabbit antithymocyte globulin, each combined with two basiliximab doses

**DOI:** 10.3389/fped.2026.1699921

**Published:** 2026-05-12

**Authors:** Gaetano Ciancio, Jeffrey J. Gaynor, Mahmoud Morsi, Angel Alvarez, Matthew Gaynor, Marissa Defreitas, Jayanthi Chandar

**Affiliations:** 1 Department of Surgery, University of Miami Miller School of Medicine, Jackson Memorial Hospital, Miami, FL, United States; 2 Department of Urology, University of Miami Miller School of Medicine, Jackson Memorial Hospital, Miami, FL, United States; 3 Miami Transplant Institute, University of Miami Miller School of Medicine, Jackson Memorial Hospital, Miami, FL, United States; 4 Divison of Pediatric Nephrology, Department of Pediatrics, University of Miami Miller School of Medicine, Jackson Memorial Hospital, Miami, FL, United States

**Keywords:** basiliximab, corticosteroid avoidance, dual-induction therapy, pediatric kidney transplantation, rabbit antithymocyte globulin, reduced tacrolimus dosing, retrospective cohort study

## Abstract

**Background:**

We retrospectively compared two distinct dual-induction strategies in pediatric kidney transplantation.

**Methods:**

Patients transplanted at our center prior to 15 February 2020 were scheduled to receive three doses of rabbit antithymocyte globulin (rATG; 1.0 mg/kg/dose) along with two doses of basiliximab (*N* = 42), while patients transplanted thereafter were scheduled to receive a single dose of rATG with two doses of basiliximab (*N* = 50). Both intent-to-treat (ITT) groups were scheduled to receive reduced tacrolimus dosing, mycophenolate acid, and early corticosteroid withdrawal. The two primary endpoints were first biopsy-proven acute rejection (BPAR) and any viral viremia. In allowing for a similar follow-up period, all clinical outcomes were compared during the first 24 months (mo) post-transplant. In addition, multivariable analyses were performed adjusting for the propensity to belong to the single-dose ITT rATG group.

**Results:**

Median follow-up in both groups was 24 mo post-transplant. In the single-dose ITT rATG group, a higher proportion of patients developed a first BPAR [*P* = 0.10; 34.0% (17/50) vs. 21.4% (9/42)], while a lower proportion developed any viral viremia [*P* = 0.08; 28.0% (14/50) vs. 47.6% (20/42)]. Two baseline variables were associated with a significantly higher propensity to belong to the single-dose ITT rATG group: older recipient age (*P* = 0.02) and longer WIT (*P* = 0.02). After adjusting for the propensity score in multivariable analyses, the single-dose ITT rATG group remained associated with a higher first BPAR rate (*P* = 0.06) but was no longer associated with the rate of viral viremia (*P* = 0.30). Estimated graft survival at 24 mo post-transplant (combined across both groups) was 92.9% ± 2.8%.

**Conclusions:**

A lower rate of first BPAR but a somewhat higher rate of viral infection was observed in the three-dose ITT rATG group. These study results support the use of tailored induction protocols to optimize effective immunosuppression while minimizing adverse effects in pediatric kidney transplantation.

## Introduction

A therapeutic strategy following pediatric kidney transplantation has included the use of single-agent induction, primarily with either the lymphodepleting polyclonal antibody rabbit antihuman thymocyte globulin (rATG) or the non-depleting anti-interleukin-2 receptor (anti-CD25) agent basiliximab, and a maintenance regimen that primarily includes reduced tacrolimus dosing (rTd) and maximized use of a non-nephrotoxic, antiproliferative drug, specifically an inosine monophosphate dehydrogenase (IMPDH) inhibitor (1). The goal of this therapeutic strategy is to minimize the incidence of acute and chronic rejection, as well as long-term calcineurin inhibitor (CNI) nephrotoxicity, thereby improving long-term graft and patient survival.

It is also known that rATG contains antibodies targeting a wide variety of peripheral blood mononuclear cell epitopes, triggering B-cell and plasma cell apoptosis and possibly exerting a protective effect against preservation injury and chronic rejection (2, 3). The use of rATG has also been shown to promote the development of regulatory T cells (T-regs) post-transplant (4), and combining anti-CD25 with rATG has been shown to more effectively delay the return of peripheral blood CD25+ cells (5). We and others have previously demonstrated that a dual-induction strategy combining lower-dose rATG with an anti-CD25 agent yields highly favorable outcomes in terms of BPAR and graft survival in adult kidney transplant recipients (6–10). Combined with this dual-induction strategy, early corticosteroid withdrawal (typically at or shortly after hospital discharge) was also employed at our center to minimize its well-known long-term side effects while maintaining favorable graft and patient survival (6, 7).

In pediatric kidney transplantation, dual-induction protocols that include planned early corticosteroid withdrawal have been routinely utilized at our center; as early as 2015, this approach included three rATG doses (1.0 mg/kg/dose) combined with two standard basiliximab doses administered during the first week post-transplant, with both agents started intraoperatively at transplant (11). In fact, during 2012–2014, our initial dual-induction protocol allowed for 3–5 rATG induction doses in addition to two standard basiliximab doses (12, 13). Starting in February 2020, the induction protocol at our center was modified to include a single rATG dose (1.0 mg/kg) in combination with two standard basiliximab doses to reduce the risk of viral infections while maintaining a low risk of acute/chronic rejection.

In this study, we specifically wanted to compare (in a retrospective, non-randomized fashion) our single-center results in using these two distinct dual-induction strategies: an earlier transplanted group scheduled to receive three rATG doses (combined with two basiliximab doses) and a more recently transplanted group scheduled to receive a single rATG dose (combined with two basiliximab doses). The main purpose of this study was to compare the effectiveness of these two dual-induction strategies in achieving favorable rates of acute rejection, viral infection, and graft loss, in conjunction with rTd, full-dose IMPDH inhibitor therapy, and corticosteroid avoidance. The results of this retrospective comparison are presented here.

## Materials and methods

### Study population and design

Between January 2015 and December 2023, 92 pediatric recipients (age <19 years) who underwent kidney-alone transplantation from either deceased donors (DDs) or non-HLA identical living donors (LDs) were included in this study. These 92 kidney transplants were performed by two transplant surgeons using surgical modifications (as previously reported) to the conventional kidney transplant technique (14, 15). Under these surgical modifications, no initial ureteral stent placement or surgical drainage was used.

This non-randomized, retrospective cohort study was approved by the Institutional Review Board at the University of Miami (IRB #20140129) and was conducted in accordance with the ethical principles (as revised in 2013) of the Declaration of Helsinki. All patients (or their guardians) provided written informed consent prior to transplantation.

This study was specifically designed to compare two intent-to-treat (ITT) induction groups: the first group (patients transplanted prior to 15 February 2020) was scheduled to receive three doses of rATG (thymoglobulin; 1.0 mg/kg/dose), while the second group (patients transplanted on or after 15 February 2020) was scheduled to receive a single dose of rATG (thymoglobulin; 1.0 mg/kg/dose). An ITT approach was utilized, with all patients analyzed according to their assigned or planned induction group. This approach, commonly used in prospective randomized clinical trials, helps avoid treatment selection bias, as a patient's clinical status at or immediately after transplantation could affect the number of rATG induction doses actually received by the patient.

### Immunosuppression

The first rATG dose was scheduled to be administered intraoperatively, with two additional equivalent doses scheduled on days 2–4 post-transplant in the three-dose group. For both ITT groups, the basiliximab induction protocol specified that patients weighing <35 kg pretransplant would receive two 10 mg doses, while those weighing ≥35 kg would receive two 20 mg doses. The first dose of basiliximab (10 or 20 mg; Simulect) was administered intraoperatively, with the second dose given 3–4 days later. In addition, in both ITT groups, the induction protocol specified that patients who were crossmatch-positive, highly sensitized, or had specific causes of end-stage renal disease (e.g., primary focal segmental glomerulosclerosis) were eligible to receive up to one 375 mg/m^2^ dose of rituximab as part of induction therapy.

Oral tacrolimus was introduced once the serum creatinine level was ≤3 mg/dL. It was initiated at 0.1 mg/kg twice daily after renal function had improved, with a target (12-h) trough level of 4–8 ng/mL. Enteric-coated mycophenolate sodium (EC-MPS) dosing was introduced on postoperative day 2 at a target dose of 600 mg/m^2^ twice daily. In some patients, mycophenolate mofetil (MMF) was used instead of EC-MPS (due to physician preference or insurance coverage). Dose adjustments were performed according to white blood cell count, gastrointestinal tolerance, and blood level(s) of any viral viremia (CMV, BK, EBV, etc.). Any withholding of EC-MPS (or MMF) for a minimum of 1 month was documented, along with the reasons. In addition, patients could be switched from an IMPDH inhibitor (due to poor tolerability or persistent viral viremia) to another maintenance agent, such as sirolimus, everolimus, or azathioprine. Methylprednisolone was administered intravenously at 10 mg/kg/day (maximum 500 mg/day) for 3 days postoperatively, followed by scheduled early withdrawal during the first postoperative week or shortly after hospital discharge (i.e., corticosteroid avoidance). Daily oral prednisone could be reinstated in patients who developed *de novo* donor-specific antibodies (DSAs) or rejection.

The scheduling of non-immunosuppressive adjunctive therapy was essentially the same as our previously described protocols (6, 7). For example, regarding cytomegalovirus (CMV) prophylaxis, all patients received intravenous ganciclovir for 3 days immediately post-transplant, followed by daily valganciclovir orally for 3 mo, with dosing adjusted based on renal function. In donor CMV Ig+/recipient CMV Ig− combinations, treatment was extended to 6 mo postoperatively. In patients who developed rejection requiring steroids or antilymphocyte therapy, intravenous ganciclovir or valganciclovir was reinstituted. Pneumocystis prophylaxis with trimethoprim-sulfamethoxazole was also administered for a minimum of 12 mo (6, 7).

Of note, all DD organs were immediately placed in a LifePort renal preservation machine with Kidney Perfusion Solution upon arrival at our center (16).

Tacrolimus trough levels were routinely measured for each patient using a whole-blood immunoassay, with blood samples taken twice weekly during the first month, once weekly during 2–3 months post-transplant, monthly for the next 6 mo, and then every 2 mo thereafter. Viral viremia (CMV, BK, and EBV) levels were also routinely monitored for each patient, with labs scheduled less often than those used to measure tacrolimus trough levels. Delayed graft function (DGF) was defined as the need for dialysis during the first week post-transplant, while slow graft function (SGF) was defined as a decrease in serum Cr of <0.5 mg/dL during the first 24 h post-transplant.

### Outcome variables

All patients were monitored for the incidence of biopsy-proven acute rejection (BPAR; also known as acute T-cell-mediated rejection), chronic allograft injury (CAI; interstitial fibrosis/tubular atrophy), any viral viremia (CMV, BK, EBV, etc.), surgical complications, infections, renal function [assessed via serum creatinine and estimated glomerular filtration rate (eGFR) using the updated Schwartz et al. equation] (17), new-onset diabetes mellitus after transplantation (NODAT), graft loss, and death.

Banff criteria were used to determine the severity of rejection and CAI (18), with BPAR requiring both clinical indication and treatment of the episode. Cases of biopsy-proven antibody-mediated rejection (AMR) were also identified (19), along with clinical documentation of the incidence of *de novo* DSA+. All kidney transplant biopsies were interpreted by experienced transplant pathologists at our center. Viral viremia (CMV, BK, EBV, etc.) was defined as viral replication exceeding 1,000 copies/mL by real-time polymerase chain reaction (12). Graft loss was defined as either the re-establishment of long-term dialysis (death-censored graft failure, DCGF) or death (i.e., death with a functioning graft, DWFG). NODAT was defined as the use of insulin or oral antihyperglycemic agents for ≥30 days in patients without a preoperative history of diabetes mellitus. Warm ischemia time (WIT) was defined as the time interval between removal of the donor kidney from machine perfusion (for DD kidneys) or ice (for LD kidneys) for implantation and the restoration of blood flow (i.e., reperfusion after vascular anastomosis).

Finally, it should be noted that although this study was designed and performed as a non-randomized retrospective cohort study, baseline and clinical outcome data for these patients were prospectively recorded in our hospital-specific and transplant-specific databases. Data retrieval was performed retrospectively.

### Statistical analysis

The primary goal of this study was to compare clinical outcomes, using an ITT approach, between pediatric patients scheduled to receive three rATG induction doses (patients transplanted prior to 15 February 2020) and those scheduled to receive a single rATG induction dose (patients transplanted on or after 15 February 2020). Both ITT groups were also scheduled to receive two basiliximab induction doses (as described above).

Patients in this study were transplanted between January 2015 and December 2023, with the last follow-up date being 31 December 2024, allowing for a minimum of 12 mo of post-transplant follow-up. Since the three-dose ITT rATG group was transplanted earlier, we wanted to perform all comparisons based on patients having similar follow-up periods in both ITT groups. Thus, all clinical outcomes were compared based on events occurring within the first 24 mo post-transplant (i.e., all clinical events occurring beyond 24 mo post-transplant were treated as censored observations).

Two primary clinical outcomes were compared between the two ITT groups during the first 24 mo post-transplant: the development of a first BPAR and the occurrence of any viral viremia. Secondary outcomes included the development of a second BPAR, acute AMR, *de novo* DSAs, “overt” non-adherence (defined as abrupt discontinuation or consistent failure to take prescribed immunosuppressive medications) (20, 21), as well as the initiation of maintenance corticosteroids (for at least 3 mo), development of CMV, BK, and EBV viremia, occurrence of a first and second infection requiring hospitalization, development of NODAT, eGFR, DCGF, and DWFG.

Means and standard errors (SEs) were calculated for all continuous (baseline and outcome) variables, along with their medians and ranges. For categorical variables, the proportions of patients with selected characteristics were determined. Comparisons of mean values between the two ITT groups were performed using standard *t*-tests, while comparisons of categorical variables were performed using Pearson’s (uncorrected) chi-squared tests. Finally, time-to-event variables between the two ITT groups were compared using log-rank tests. Of note, no adjustments were made for performing multiple comparisons.

Multivariable analyses were also performed to compare clinical outcomes between the two ITT groups. First, stepwise logistic regression was used to assess the likelihood of belonging to the single-dose ITT rATG group (i.e., patients transplanted on or after 15 February 2020), and a propensity score for each patient was determined from the selected multivariable logistic regression model. Second, stepwise Cox regression analyses were performed to identify significant multivariable predictors of the development of first BPAR and any viral viremia. Stepwise linear regression was also utilized to determine the significant multivariable predictors of eGFR at both 12 and 24 mo post-transplant. As a statistical note, “stepwise regression” implies that no significant predictor variables are assumed to exist *a priori*; only those found to be significant during the stepwise regression process are selected and retained in the multivariable model. A multivariable assessment of the prognostic effect of the ITT group was then performed within each multivariable model, controlling for the effects of significant multivariable predictors and the propensity to belong to the single-dose ITT rATG group.

Graphical displays of time-to-event outcomes, including the development of first BPAR, occurrence of any viral viremia, and graft survival during the first 24 mo post-transplant, were performed using the Kaplan–Meier technique. Kaplan–Meier curves were also used to illustrate the effects of prognostic variables on these time-to-event outcomes.

Finally, we determined the prognostic impact of developing a first BPAR (modeled as a time-dependent zero–one covariate) on the subsequent hazard of developing any viral viremia (i.e., testing the prognostic effect of first BPAR development on the subsequent risk of developing any viral viremia). Conversely, we evaluated the prognostic impact of developing any viral viremia (as a time-dependent zero–one covariate) on the subsequent hazard of developing a first BPAR (i.e., testing the prognostic effect of any viral viremia development on the subsequent risk of developing a first BPAR). These analyses were performed using the Cox proportional hazards model (22).

## Results

### Univariable comparisons between the two ITT rATG groups

Associations between selected baseline variables, induction actually received, and clinical outcome with the two scheduled (ITT) rATG induction groups are presented in Tables 1–3, respectively. Significant univariable associations were observed for recipient age (and thus, recipient height and recipient weight) and WIT. Specifically, mean recipient age was significantly higher in the more recently transplanted single-dose ITT rATG group (*P* = 0.02), with 35.7% (15/42) of patients in the three-dose ITT rATG being ≥13 years of age at transplant compared with 62.0% (31/50) in the single-dose ITT rATG group (*P* = 0.01) (note: the median age at transplant for the whole cohort of 92 patients was 13 years). Mean WIT was also significantly longer in the more recently transplanted single-dose ITT rATG group (31.8 ± 0.8 min) compared with the three-dose ITT rATG group (29.1 ± 0.9 min) (*P* = 0.03), likely reflecting that the more experienced surgeon performed most of the earlier transplants (further details not shown). No other significant differences in the frequency distributions of baseline variables were observed between the two ITT rATG groups (Table 1).

**Table 1:** Tests of associations of selected baseline variables with the scheduled (ITT) rATG induction: three doses vs. one dose (of 1 mg/kg/day).

	Percentage with characteristic for categorical variables; mean ± SE for continuous variables		
	Intent-to-treat rATG induction		
Baseline variable	Three doses (*N* = 42)	One dose (*N* = 50)	*P*-value^a^
Recipient age (years)	10.3 ± 0.8 (*N* = 42)	12.8 ± 0.7 (*N* = 50)	0.02
Recipient age ≥13 years, the overall median	35.7% (15/42)	62.0% (31/50)	0.01
Male recipient	66.7% (28/42)	62.0% (31/50)	0.64
Recipient race/ethnicity			0.87
African-American (non-Hispanic)	35.7% (15/42)	32.0% (16/50)	
Hispanic	40.5% (17/42)	46.0%(23/50)	
Caucasian (non-Hispanic)/other^b^	23.8% (10/42)	22.0% (11/50)	
Recipient height (cm)	127.5 ± 4.4 (*N* = 42)	140.5 ± 4.0 (*N* = 50)	0.03
Recipient weight (kg)	32.9 ± 3.0 (*N* = 42)	42.6 ± 3.0 (*N* = 50)	0.03
Recipient BMI (kg/m^2^)	18.4 ± 0.6 (*N* = 42)	20.2 ± 0.7 (*N* = 50)	0.07
Retransplant recipient	0.0% (0/42)	6.0% (3/50)	0.11
Preemptive transplant	23.8% (10/42)	32.0% (16/50)	0.38
Pretransplant time on dialysis (mo)^c^	19.9 ± 3.0 (*N* = 42)	19.7 ± 3.4 (*N* = 50)	0.98
Cause of ESRD			0.69
Acquired	33.3% (14/42)	42.0% (21/50)	
Congenital	61.9% (26/42)	54.0% (27/50)	
Unknown	4.8% (2/42)	4.0% (2/50)	
DD recipient	69.0% (29/42)	60.0% (30/50)	0.37
# Donor arteries ≥2	9.5% (4/42)	20.0% (10/50)	0.16
Donor age (years)	27.9 ± 1.5 (*N* = 42)	31.3 ± 1.7 (*N* = 50)	0.15
Donor age ≥35 years	26.2% (11/42)	44.0% (22/50)	0.08
CIT (h)	16.6 ± 1.9 (*N* = 42)	14.8 ± 1.8 (*N* = 50)	0.51
CIT ≥18 h	57.1% (24/42)	52.0% (26/50)	0.62
WIT (min)	29.1 ± 0.9 (*N* = 42)	31.8 ± 0.8 (*N* = 50)	0.03
KDPI ≥20% (among DD recipients)	24.1% (7/29)	43.3% (13/30)	0.12
Pretransplant cPRA (%)	5.3 ± 2.2 (*N* = 42)	7.5 ± 3.0 (*N* = 50)	0.55
Pretransplant DSA+	14.3% (6/42)	12.0% (6/50)	0.75

a Categorical variables were compared using Pearson’s (uncorrected) chi-squared test. Continuous variables were compared using the *t*-test. Time-to-event variables were compared by the log-rank test.

b Four patients that were either Asian or Middle Eastern (one in the three-dose ITT rATG group and three in the single-dose ITT rATG group) were combined with the white (non-Hispanic) group.

c Pretransplant time on dialysis (mo) was scored as 0 for patients who underwent preemptive transplantation.

**Table 2:** Tests of associations of induction actually received with the scheduled (ITT) rATG induction: three doses vs. one dose (of 1 mg/kg/day).

	Percentage with characteristic for categorical variables; mean ± SE for continuous variables		
	Intent-to-treat rATG induction		
Induction actually received	Three doses (*N* = 42)	One dose (*N* = 50)	*P*-value^a^
Received the planned rATG induction^b^	76.2% (32/42)	74.0% (37/50)	0.81
Received two doses of basiliximab^c^	97.6% (41/42)	98.0% (49/50)	0.90
Received induction with rituximab^d^	7.1% (3/42)	18.0% (9/50)	0.12

a Categorical variables were compared using Pearson’s (uncorrected) chi-squared test. Continuous variables were compared using the *t*-test. Time-to-event variables were compared by the log-rank test.

b Among the 10 patients in the three-dose ITT rATG group who did not receive the planned rATG induction, two patients received 0 doses due to immune deficiency/low WBC (*N* = 1) and experiencing an immediate allergic reaction to receiving rATG (*N* = 1). Four patients in the three-dose ITT rATG group received only one rATG dose due to the patient having a high-grade fever (*N* = 1), high-grade fever combined with suspected peritonitis (*N* = 1), pretransplant history of infection (*N* = 1), and donor-derived infection (*N* = 1). Two patients in the three-dose ITT rATG group received only two rATG doses due to donor-derived infection (*N* = 1) and hypotension after receiving the second dose (*N* = 1). One patient in the three-dose ITT rATG group received four rATG doses for an unspecified reason. Finally, one patient in the three-dose ITT rATG group received five rATG doses due to experiencing SGF and a delay in starting TAC. Among the 13 patients in the single-dose ITT rATG group who did not receive the planned rATG induction, two patients received no dose due to low CD4 count and being non-immune to varicella (*N* = 1) and a low CD4 count (*N* = 1). One patient in the single-dose ITT rATG group received less than the planned single dose of 1.0 mg/kg (roughly 0.75 mg/kg) due to cytokine release syndrome during rATG administration. Six patients in the single-dose ITT rATG group received two rATG doses due to SGF and a delay in starting TAC (*N* = 3), SGF and a slowly decreasing serum creatinine level (*N* = 2), and high sensitization (*N* = 1). Four patients in the single-dose ITT rATG group received three rATG doses due to SGF and a delay in starting TAC (*N* = 3) and high sensitization (*N* = 1).

c One patient in the three-dose ITT rATG group who weighed 17.5 kg received only one 10-mg dose of basiliximab; no reason for this protocol deviation was given. One patient in the single-dose ITT rATG group who weighed 85.8 kg experienced graft failure immediately post-transplant (on day 0) and was not given the second 20-mg dose.

d Among the three patients in the three-dose ITT rATG group who received eituximab, the indications are as follows: highly sensitized pretransplant (*N* = 2; both patients were pretransplant DSA+, with cPRA values of 33% and 81%) and immune complex crescentic glomerulonephritis as the cause of ESRD (*N* = 1). Among the nine patients in the single-dose ITT rATG group who received rituximab, the indications are as follows: highly sensitized pretransplant (*N* = 3; two of three patients were pretransplant DSA+, with cPRA values of 46%, 92%, and 98%), positive donor B-cell crossmatch  (*N* = 1), pre-existing DSA+ without high sensitization (*N* = 1, cPRA was 8%), development of *de novo* DSA+ immediately post-transplant (*N* = 1), serologically active SLE (*N* = 1), and prophylaxis against FSGS recurrence (*N* = 2).

**Table 3:** Tests of associations of clinical outcome variables with the scheduled (ITT) rATG.

	Percentage with characteristic for categorical variables; mean ± SE for continuous variables		
	Intent-to-treat rATG induction		
Clinical outcome variable	Three doses (*N* = 42)	One dose (*N* = 50)	*P*-value^a^
DGF occurrence	2.4% (1/42)	0.0% (0/50)	0.27
Developed a first BPAR (first 24 mo)^b^	21.4% (9/42)	34.0% (17/50)	0.10
Developed a first BPAR of grade borderline or IA (first 24 mo)^b^	11.9% (5/42)	26.0% (13/50)	0.05
Developed a second BPAR (first 24 mo)	2.4% (1/42)	8.0% (4/50)	0.19
Developed *de novo* DSA (first 24 mo)	26.2% (11/42)	18.0% (9/50)	0.49
Developed acute AMR (first 24 mo)^b^	14.3% (6/42)	10.0% (5/50)	0.74
Developed overt non-adherence (first 24 mo)^b^	2.4% (1/42)	6.0% (3/50)	0.32
Placed on maintenance corticosteroids (for at least 3 mo) (during the first 24 mo)^c^	35.7% (15/42)	40.0% (20/50)	0.67
Developed any viral viremia (first 24 mo)^d^	47.6% (20/42)	28.0% (14/50)	0.08
Developed CMV viremia (first 24 mo)^e^	19.0% (8/42)	12.0% (6/50)	0.39
Developed BK viremia (first 24 mo)^f^	31.0% (13/42)	16.0% (8/50)	0.09
Developed EBV viremia (first 24 mo)^g^	11.9% (5/42)	2.0% (1/50)	0.09
Developed a first infection requiring hospitalization (first 24 mo)	47.6% (20/42)	36.0% (18/50)	0.30
Developed a second infection requiring hospitalization (first 24 mo)	16.7% (7/42)	16.0% (8/50)	0.96
Developed NODAT (first 24 mo)	0.0% (0/42)	4.0% (2/50)	0.19
eGFR at 1 mo post-transplant (mL/min/1.73m^2^)	92.0 ± 4.8 (*N* = 42)	75.9 ± 4.6 (*N* = 49)	0.02
eGFR at 6 mo post-transplant (mL/min/1.73 m^2^)	84.3 ± 4.5 (*N* = 42)	71.5 ± 3.9 (*N* = 49)	0.03
eGFR at 12 mo post-transplant (mL/min/1.73 m^2^)	78.5 ± 3.4 (*N* = 42)	65.3 ± 4.1 (*N* = 48)	0.02
eGFR at 24 mo post-transplant (mL/min/1.73 m^2^)	71.4 ± 3.7 (*N* = 32)	62.4 ± 4.5 (*N* = 23)	0.13
Developed (death-censored) graft failure (first 24 mo)^h^	2.4% (1/42)	10.0% (5/50)	0.13
Death with a functioning graft (first 24 mo)	0.0% (0/42)	0.0% (0/50)	1.00

a Categorical variables were compared using Pearson’s (uncorrected) chi-squared test. Continuous variables were compared using the *t*-test. Time-to-event variables were compared by the log-rank test.

b Grades of first BPAR for the nine patients in the three-dose ITT rATG group were as follows: borderline in two cases, IA in three cases, IB in two cases, IIA in one case, and III in one case. Grades of first BPAR for the 17 patients in the single-dose ITT rATG group were as follows: borderline in eight cases, IA in five cases, IB in two cases, IIA in one case, and IIB in one case. Antilymphocyte treatment (with rATG) of first BPAR was used in two of nine cases in the three-dose ITT rATG group (in one grade IB case, and in one grade IIA case). Antilymphocyte treatment (with rATG) of first BPAR was used in three of 17 cases in the single-dose ITT rATG group (in one grade IB case, in one grade IIA case, and in one grade IIB case). Of note, in each of the 11 acute AMR cases that occurred, a first BPAR occurred simultaneously with the development of acute AMR. Finally, in the three-dose ITT rATG group, overt non-adherence occurred prior to first BPAR occurrence in one of one case. In the single-dose ITT rATG group, overt non-adherence occurred prior to first BPAR occurrence in two of three cases and prior to second BPAR occurrence in one of three cases.

c Among the 15 patients in the three-dose ITT rATG group who were placed on maintenance corticosteroids for at least 3 months post-transplant, the reasons were as follows: five following a first BPAR; five due to mycophenolate acid being withheld (discontinued or reduced in dose) because of leukopenia/neutropenia (in three cases), viral infection (in one case), or GI symptoms (in one case); two to optimize immunosuppression (mainly due to difficulty achieving more optimal/higher TAC trough levels); one following initial hospital discharge due to high sensitization; one following initial hospital discharge due to not receiving any rATG induction (secondary to an allergic reaction); and one for an unknown reason following initial hospital discharge. Among the 20 patients in the single-dose ITT rATG group who were placed on maintenance corticosteroids for at least 3 months post-transplant, the reasons were as follows: five following a first BPAR; seven due to mycophenolate acid being withheld (discontinued or reduced in dose) because of leukopenia/neutropenia (in two cases), viral infection (in three cases), or GI symptoms (in two cases); three to optimize immunosuppression (mainly due to difficulty achieving more optimal/higher TAC trough levels); three following initial hospital discharge due to high sensitization; one due to the patient having FSGS; and one following initial hospital discharge to avoid development of adrenal insufficiency.

d Among the 20 patients in the three-dose ITT rATG group who developed a viral viremia during the first 24 months post-transplant, the types of viruses detected were as follows: CMV viremia only (*N* = 4), EBV viremia subsequently followed by CMV viremia (*N* = 1), BK viremia subsequently followed by CMV viremia (*N* = 1), CMV viremia subsequently followed by EBV viremia and then subsequently followed by BK viremia (*N* = 1), CMV viremia subsequently followed by BK viremia (*N* = 1), BK viremia only (*N* = 8), BK viremia subsequently followed by EBV viremia (*N* = 1), BK viremia subsequently followed by adenovirus viremia (*N* = 1), and EBV viremia only (*N* = 2). Among the 14 patients in the single-dose ITT rATG dose group who developed a viral viremia during the first 24 mo post-transplant, the types of viruses detected were as follows: CMV viremia only (*N* = 2), CMV viremia subsequently followed by BK viremia (*N* = 1), CMV viremia and BK viremia simultaneously (*N* = 1), CMV viremia and EBV viremia simultaneously (*N* = 1), CMV viremia and adenovirus viremia simultaneously (*N* = 1), BK viremia only (*N* = 6), and parvovirus viremia only (*N* = 2).

e Overall, the median time-to-first detection of CMV viremia for these 14 cases was 6.1 [range: 1.8–14.9] months post-transplant. The median CMV viremia count at the time of first detection in 11 of 14 cases was 6,296 [range: 1,421–31,765]; the CMV viremia count at the time of first detection was unavailable in three of 14 cases, as these three patients were diagnosed with CMV viremia at another center. In 12 of 14 cases, the dose of Valcyte was either increased or restarted; in nine of 14 cases, the dose of MMF (or EC-MPS) was either reduced, temporarily withheld, or discontinued.

f Overall, the median time-to-first detection of BK viremia for these 21 cases was 6.6 [range: 0.9–19.0] months post-transplant. The median BK viremia count at the time of first detection for these 21 cases was 18,708 [range: 1,150–610,000]. In eight of 21 cases, the patient received treatment with Leflunomide; in 13 of 21 cases, the dose of MMF (or EC-MPS) was either reduced, temporarily withheld, or discontinued. In one case, tacrolimus was temporarily withheld while the patient received Leflunomide; in four of 21 cases, no treatment for BK viremia or any changes in immunosuppression were made (observation only). Also of note, in one of 21 cases, a patient (in the single-dose ITT rATG group) was also diagnosed with biopsy-proven BK nephropathy at 3.3 months post-transplant.

g Overall, the median time-to-first detection of EBV viremia for these six cases was 11.0 [range: 1.6–23.0] months post-transplant. The median EBV viremia count at the time of first detection in four of six cases was 1,335 [range: 257–2,044]; the EBV viremia count at the time of first detection was unavailable in two of six cases. In three of six cases, treatment with Valcyte was restarted; in five of six cases, the dose of MMF (or EC-MPS) was either reduced, temporarily withheld, or discontinued.

h In the three-dose ITT rATG group, median follow-up among 41 patients who were alive with a functioning graft at either last follow-up or 24 mo post-transplant (whichever occurred first) was 24.0 [range: 12.0–24.0] months post-transplant; 37 of 41 patients were followed through 24 mo post-transplant (i.e., four patients were lost to follow-up between 12 and 14.5 mo post-transplant). In the single-dose ITT rATG group, median follow-up among 45 patients who were alive with a functioning graft at either last follow-up or 24 mo post-transplant (whichever occurred first) was also 24.0 [range: 12.4–24.0] months post-transplant. While none of 45 these patients were lost to follow-up during the first 24 mo post-transplant, 35 of 45 and 24 of 45 of these patients were followed through 18 and 24 mo post-transplant, respectively. The cause of graft failure for the single patient who experienced graft failure in the three-dose ITT rATG group during the first 24 mo post-transplant was overt non-adherence (at 15.4 mo post-transplant). Causes of graft failure for the five patients who experienced graft failure in the single-dose ITT rATG group during the first 24 mo post-transplant included: renal vein thrombosis (*N* = 1, at day 0 post-transplant), primary disease (rapidly progressing glomerulonephritis) recurrence (*N* = 1, at 12.0 mo post-transplant), CAI and chronically high parvovirus infection (*N* = 1, at 13.3 mo post-transplant), and overt non-adherence (*N* = 2, at 15.6 and 18.3 mo post-transplant). Of note, the single patient in the three-dose ITT rATG group who experienced graft failure due to overt non-adherence occurring at 15.4 mo post-transplant died of a cardiovascular event at 6 days following the graft failure date.

In terms of induction therapy actually received, the proportions of patients in the two ITT groups who received their planned rATG and basiliximab induction doses were similar, with 74.0% (37/50) of patients in the single-dose rATG ITT group and 76.2% (32/42) in the three-dose ITT rATG group receiving their planned rATG induction (*P* = 0.81, Table 2). Of note, in the single-dose ITT rATG group, 6.0% (3/50) of patients received <1 complete dose, while 20.0% (10/50) of patients received >1 complete dose. Conversely, 19.0% (8/42) of patients in the three-dose ITT rATG group received <3 complete doses, while 4.8% (2/42) received >3 complete doses. Reasons for not receiving the planned rATG induction are provided in footnote b of Table 2. There was also no significant difference in the proportion of patients who received rituximab as part of induction therapy (Table 2).

In terms of clinical outcome variables, Table 3 shows that patients in the single-dose ITT rATG group trended to have a higher hazard of developing a first BPAR during the first 24 mo post-transplant [34.0% (17/50) vs. 21.4% (9/42) in the three-dose ITT rATG group; *P* = 0.10]. Notably, a significantly higher hazard of developing a first BPAR of grade ≤ IA (borderline or IA) was observed in the single-dose ITT rATG group [26.0% (13/50) vs. 11.9% (5/42) in the three-dose ITT rATG group; *P* = 0.05]. Conversely, there was a trend toward a lower incidence of viral viremia in the single-dose ITT rATG group compared with the three-dose ITT rATG group [28.0% (14/50) vs. 47.6% (20/42); *P* = 0.08]. While the incidence of CMV viremia did not appear to differ between the two ITT groups (*P* = 0.39), the incidence rates of BK and EBV viremia appeared to be higher in the three-dose ITT rATG group (*P* = 0.09 each). Finally, mean eGFR at 1, 6, and 12 mo post-transplant was significantly lower in the single-dose ITT rATG group (*P* = 0.02, 0.03, and 0.02, respectively). No other significant differences between the two ITT rATG groups were observed for outcome variables, including the development of *de novo* DSAs, acute AMR, overt non-adherence, first (or second) infections requiring hospitalization, NODAT, and death-censored graft failure (Table 3). No deaths with a functioning graft were observed in either group during the first 24 mo post-transplant.

Table 3 shows that the observed proportion of patients who developed death-censored graft failure was 2.4% (1/42) in the three-dose ITT rATG group and 10.0% (5/50) in the single-dose rATG groups (*P* = 0.13, log-rank test). Causes of graft failure are listed in footnote h of Table 3. Of note, in the three-dose ITT rATG group, 37 of 41 patients who did not experience graft failure were followed through 24 mo post-transplant (with four patients lost to follow-up between 12 and 14.5 mo post-transplant). In the single-dose ITT rATG group, none of the 45 patients who did not experience graft failure were lost to follow-up during the first 24 mo post-transplant; however, 35 of 45 and 24 of 45 patients were followed through 18 and 24 mo post-transplant, respectively.

### Propensity to belong to the single-dose ITT rATG group

Two baseline variables were identified via stepwise logistic regression as independently associated with a higher likelihood of belonging to the single-dose ITT rATG group (Table 4): older recipient age (years) and longer WIT (min) (both treated as continuous variables). Once these variables were included, no other variables contributed additional predictive value (*P* > 0.05). A propensity score for each patient was then calculated based on the logistic model coefficients derived from these two selected variables.

**Table 4:** Stepwise logistic regression results (multivariable model obtained) for the likelihood of being scheduled to receive one induction rATG dose (1.0 mg/kg) (50/92).

	Univariable	Multivariable	Model^a^
Baseline variable	*P*-value	*P*-value	Coeff ± SE
Older recipient age (years)	0.02	0.02 [√]	0.101 ± 0.043
Longer WIT (min)	0.03	0.02 [√]	0.097 ± 0.042

a Baseline variables selected for the logistic regression model are defined as follows (shown by order of selection): recipient age (years) (continuous variable) and warm ischemia time (min) (continuous variable). Once these two variables were selected, no other variables contributed additional predictive value (*P* > 0.05). The intercept ± SE for this logistic model was −3.939 ± 1.458. Thus, the propensity score for the single-dose rATG induction group was determined as −3.939 + 0.101*recipient age (years) + 0.097* warm ischemia time (min).

[√] represents selection in the logistic regression model.

### Multivariable analyses of the hazard rates of developing a first BPAR and any viral viremia

**Figure 1:**
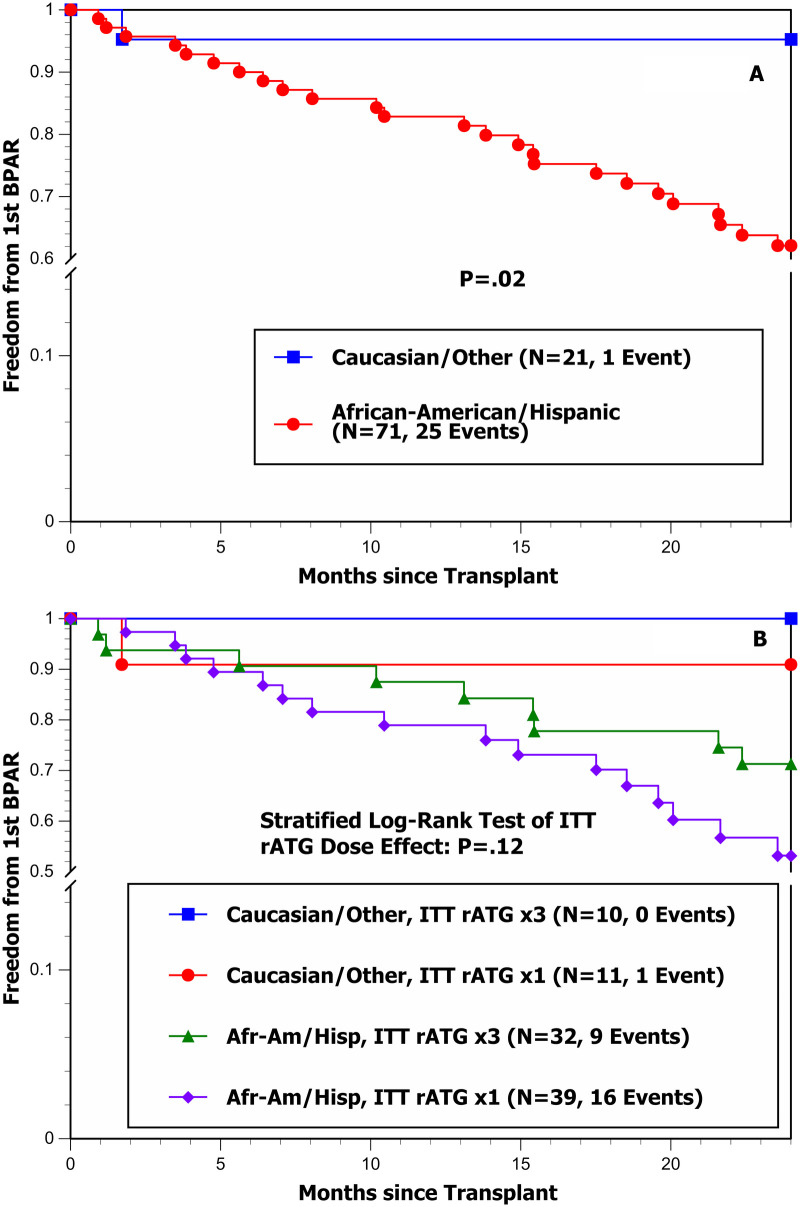
**(A)** Freedom from first BPAR by recipient race/ethnicity during the first 24 mo post-transplant. **(B)** Freedom from first BPAR by ITT rATG induction dose and recipient race/ethnicity during the first 24 mo post-transplant.

Stepwise Cox regression analysis of the hazard rate of developing a first BPAR identified one baseline variable significantly associated with a higher hazard rate: being an African-American or Hispanic recipient (*P* = 0.02) (Table 5). Once this variable was selected, no other baseline variables demonstrated additional predictive value (*P* > 0.10). This Cox model was then re-run with additional adjustment for the joint effects of single-dose ITT rATG induction and its propensity score (Table 6), and a trend toward a higher hazard of first BPAR was observed in the single-dose ITT rATG group (multivariable *P* = 0.06). These effects are graphically illustrated by the Kaplan–Meier freedom-from-first BPAR curves in Figures 1A, B. Figure 1A clearly shows the unfavorable effect of being an African-American or Hispanic recipient, with 25 of 71 patients developing a first BPAR compared with only 1 of 21 Caucasian/other recipients (e.g., freedom from first BPAR at 12 mo post-transplant was 82.9% ± 4.5% for African-American/Hispanic recipients vs. 95.2% ± 4.7% for Caucasian/other recipients). Figure 1B shows a slightly unfavorable effect of the single-dose ITT rATG group after stratification by recipient race (stratified log-rank test *P* = 0.12).

**Table 5:** Stepwise Cox regression results (multivariable model obtained) for the hazard of developing a first BPAR during the first 24 months post-transplant (26 events).

	Univariable	Multivariable model^a^	
Baseline variable	*P*-value	*P*-value	Coeff ± SE
African-American or Hispanic recipient	0.02	0.02 [√]	1.989 ± 1.020

a The single baseline variable selected for the Cox model was defined as follows: African-American or Hispanic recipient (zero–one variable) {=1 if African-American or Hispanic recipient, 0 otherwise}. Once this variable was selected, no other baseline variables contributed additional predictive value (*P* > 0.10).

[√] represents selection in the Cox model.

**Table 6:** Final Cox model for the hazard of developing a first BPAR during the first 24 mo post-transplant re-run with additional adjustment for the joint effects of single-dose ITT rATG induction and its propensity score (26 events).

	Univariable	Multivariable model^a^	
Baseline variable	*P*-value	*P*-value	Coeff ± SE
African-American or Hispanic recipient	0.02	0.02	1.989 ± 1.020
Intent to treat: single rATG induction dose	0.10	0.06	0.854 ± 0.460
Propensity score for intent to treat: single rATG induction dose	0.79	0.28	−0.228 ± 0.208

a Baseline variables that were included in this Cox model were defined as follows: Black or Hispanic recipient (zero-one variable) {=1 if African-American or Hispanic recipient, 0 otherwise}, single-dose ITT rATG Induction (zero–one variable) {=1 if single-dose ITT rATG induction, 0 otherwise}, and propensity score for single-dose ITT rATG induction [continuous variable, determined by the logistic regression model in Table 4 as −3.939 + 0.101*recipient age (years) + 0.097* warm ischemia time (min)].

Stepwise Cox regression analysis of the hazard rate of developing any viral viremia identified two baseline variables significantly associated with a higher rate: younger recipient age (multivariable *P* = 0.003) and the presence of pre-existing DSA+ (*P* = 0.02) (Table 7). Once these two variables were selected, no other baseline variables provided additional predictive value (*P* > 0.05). This Cox model was then re-run with additional adjustment for the joint effects of single-dose ITT rATG induction and its propensity score (Table 8)—the multivariable effect of single-dose ITT rATG induction was non-significant (multivariable *P* = 0.30). Further analyses by type of viral viremia found that younger recipient age was significantly associated with a higher hazard rate of developing BK viremia (*P* = 0.001), whereas the presence of pre-existing DSA+ was not (*P* = 0.36). Conversely, recipient age was not associated with the hazard rate of developing CMV viremia (*P* = 0.49), while the presence of pre-existing DSA+ was associated with a significantly higher hazard of developing CMV viremia (*P* = 0.00007).

**Table 7:** Stepwise Cox regression results (multivariable model obtained) for the hazard rate of developing any viral viremia (CMV, BK, EBV, parvovirus, and adenovirus) during the first 24 months post-transplant (34 events).

	Univariable	Multivariable model^a^	
Baseline variable	*P*-value	*P*-value	Coeff ± SE
Younger recipient age (years)	0.003	0.003 [√]	−0.095 ± 0.033
Pretransplant DSA+	0.02	0.02 [√]	0.949 ± 0.406

a Baseline variables selected for the Cox model are defined as follows (shown by order of selection): recipient age (years) (continuous variable) and pretransplant DSA+ (zero–one variable) {=1 if the recipient had pretransplant DSAs, 0 otherwise}. Once these two variables were selected, no other variables contributed additional predictive value (*P* > 0.05). Of note, while the results in Table 1 showed a borderline univariable association of single-dose ITT rATG induction with a lower hazard of developing any viral viremia during the first 24 months post-transplant (*P* = 0.08), the multivariable score test to include this variable following the inclusion of the two selected variables (as shown above) yielded *P* = 0.28. This non-significant association appears to be mainly due to the association of single-dose ITT rATG induction with older recipient age (years) (*P* = 0.02, as shown in Table 1).

[√] represents selection in the Cox model.

**Table 8:** Final Cox model for the hazard rate of developing any viral viremia (CMV, BK, EBV, parvovirus, and adenovirus) during the first 24 mo post-transplant re-run with additional adjustment for the joint effects of single-dose ITT rATG induction dose and its propensity score (34 events).

	Univariable	Multivariable model^a^	
Baseline variable	*P*-value	*P*-value	Coeff ± SE
Younger recipient age (years)	0.003	0.02	−0.091 ± 0.039
Pretransplant DSA+	0.02	0.03	0.891 ± 0.413
Single-dose ITT rATG induction	0.08	0.30	−0.394 ± 0.385
Propensity score for single-dose ITT rATG induction	0.05	0.93	0.020 ± 0.219

^a^ Baseline variables that were included in this Cox model were defined as follows: recipient age (years) (continuous variable), pretransplant DSA+ (zero–one variable) {=1 if the recipient had pretransplant DSAs, 0 otherwise}, single-dose ITT rATG induction (zero–one variable) {=1 if single-dose ITT rATG induction, 0 otherwise}, and propensity score for single-dose ITT rATG induction [continuous variable, determined by the logistic regression model in Table 4 as −3.939 + 0.101*recipient age (years) + 0.097* warm ischemia time (min)].

**Figure 2:**
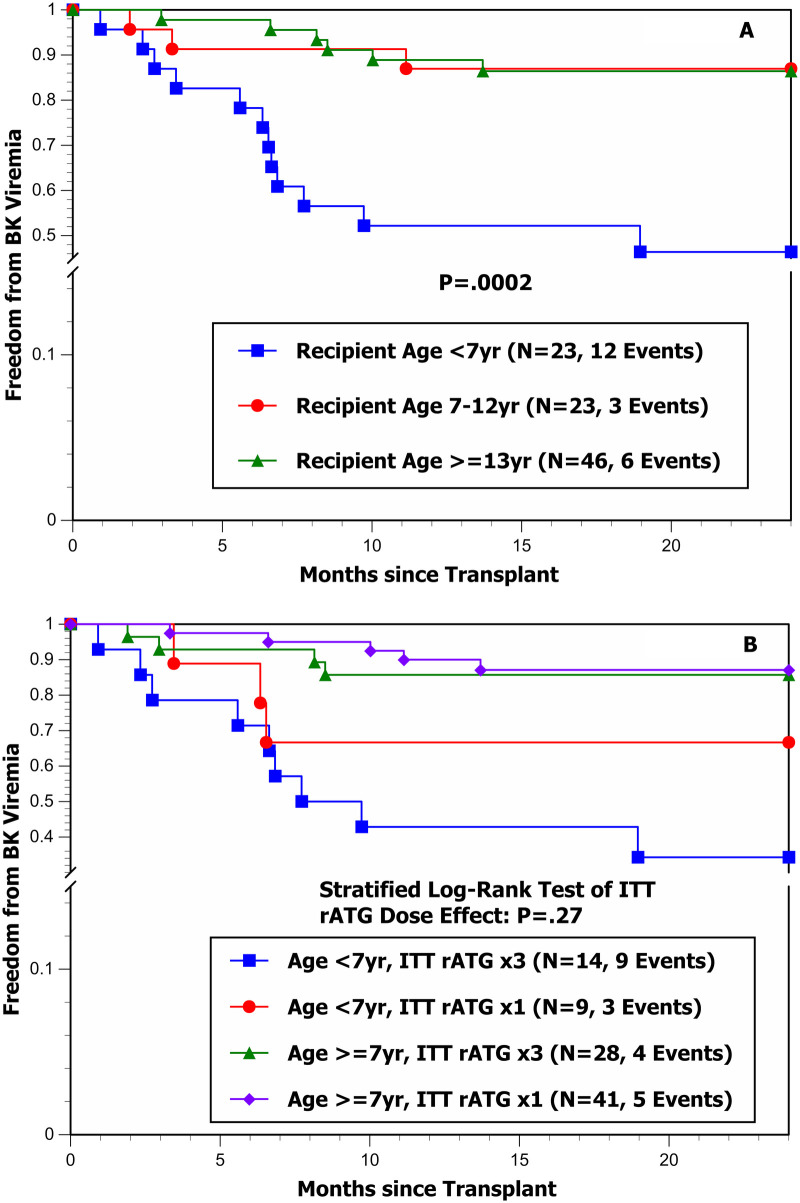
**(A)** Freedom from BK viremia by recipient age during the first 24 mo post-transplant. **(B)** Freedom from BK viremia by ITT rATG induction dose and recipient age during the first 24 mo post-transplant.

**Figure 3:**
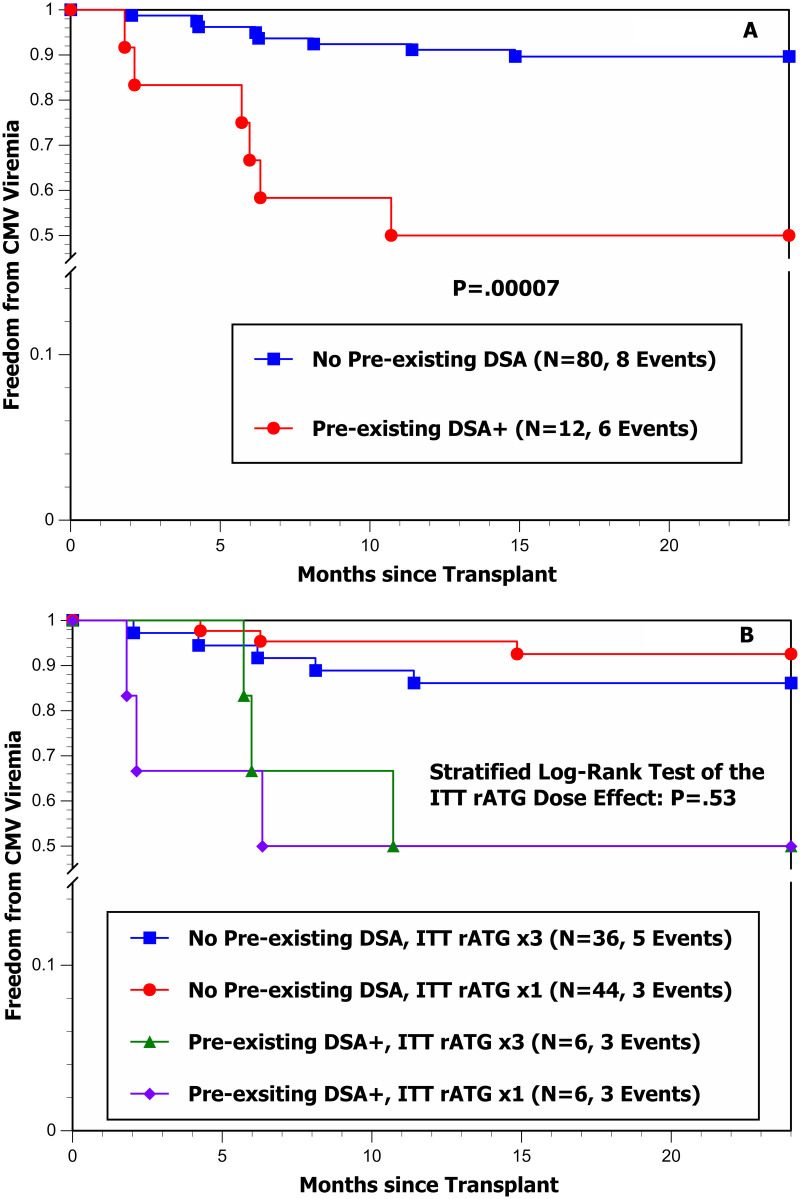
**(A)** Freedom from CMV viremia by pre-existing DSA (−/+) during the first 24 mo post-transplant. **(B)** Freedom from CMV viremia by ITT rATG induction dose and pre-existing DSA (−/+) during the first 24 mo post-transplant.

The results for BK and CMV viremia are graphically portrayed in Figures 2A,B, 3A,B, respectively. Figure 2A clearly shows that the risk of BK viremia is significantly higher in younger recipients (particularly those <7 years of age); however, Figure 2B shows very little effect of ITT rATG dose on the hazard of developing BK viremia after adjusting for recipient age (<7 vs. ≥7 years; stratified log-rank test *P* = 0.27). Figure 3A clearly shows a significantly higher hazard of developing CMV viremia in patients with pre-existing DSA+; however, Figure 3B shows no effect of ITT rATG dose on the hazard of developing CMV viremia after adjusting for the presence of pre-existing DSA+ (no/yes; stratified log-rank test *P* = 0.53).

Finally, the prognostic effect of developing any viral viremia on the hazard of developing a first BPAR was assessed as a time-dependent zero–one covariate, as was the prognostic effect of developing a first BPAR on the hazard of developing any viral viremia. The univariable prognostic effect of developing any viral viremia on the hazard of developing a first BPAR was non-significant (*P* = 0.14); its multivariable effect (after adjusting for recipient race) yielded *P* = 0.19. However, different effects were observed when viremia type was considered. Specifically, while both the univariable and multivariable effects of developing BK viremia on the hazard of developing a first BPAR were clearly non-significant (*P* = 0.98 and 0.94, respectively), the development of CMV viremia was associated with a significantly higher hazard rate of subsequently developing a first BPAR (univariable *P* = 0.02; multivariable *P* = 0.02). Conversely, both the univariable and multivariable effects of developing a first BPAR on the subsequent hazard of developing any viral viremia were non-significant (univariable *P* = 0.66; multivariable *P* = 0.58, after adjusting for the effects of recipient age and pre-existing DSA+).

### Multivariable analyses of eGFR at 12 and 24 mo post-transplant

Stepwise linear regression of eGFR at 12 mo post-transplant identified five variables independently associated with a significantly lower eGFR (Table 9, in order of selection): developing a first BPAR by 12 mo post-transplant (*P* = 0.000005), taller recipient height at 12 mo (cm) (*P* = 0.00005), male recipient (*P* = 0.03), longer CIT (h) (*P* = 0.004), and older donor age (years) (*P* = 0.04). Once these five variables were selected, no other variables contributed additional predictive value (*P* > 0.05). The coefficient of multiple determination, *R*^2^, for this five-variable linear model was 0.45. Of note, the univariable and multivariable *P*-values for the development of any viral viremia by 12 mo post-transplant to enter this linear regression model were 0.68 and 0.36, respectively (indicating no significant association); this lack of significance remained true even when BK and CMV viremia were modeled separately.

**Table 9:** Stepwise linear regression results (multivariable model obtained) for eGFR at 12 months post-transplant (*N* = 90).

	Univariable	Multivariable model^a^	
Variable	*P*-value	*P*-value	Coeff ± SE
Developing a first BPAR by 12 mo	0.000009	0.000005 [√]	−30.176 ± 6.177
Taller recipient height at 12 mo (cm)	0.00004	0.00005 [√]	−0.369 ± 0.087
Male recipient	0.45	0.03 [√]	−9.927 ± 4.511
Longer CIT (h)	0.01	0.004 [√]	−0.608 ± 0.205
Older donor age (years)	0.23	0.04 [√]	−0.485 ± 0.232

a Variables selected for the linear regression model were defined as follows (shown by order of selection): developing a first BPAR by 12 months post-transplant ={1 if a first BPAR occurred by 12 months post-transplant, 0 otherwise}, recipient height (cm) at 12 months post-transplant (continuous variable), male recipient (zero–one variable) {=1 if the recipient is male, 0 otherwise}, CIT (h) (continuous variable), and donor age (years) (continuous variable). Once these variables were selected (or included), no other variables contributed additional predictive value (*P* > 0.05). The coefficient of multiple determination, *R*^2^, achieved for this five-variable linear model was 0.45. Of note, the univariable and multivariable *P*-values for developing any viral viremia by 12 months post-transplant to enter this linear regression model were 0.68 and 0.36, respectively (clearly non-significant); this non-significant finding remained true even if BK or CMV viremia were specifically modeled.

[√] represents selection (or inclusion) in the linear regression model.

The five-variable linear regression model was then re-run with additional adjustment for the joint effects of single-dose ITT rATG and its propensity score (Table 10), and a trend toward a significantly lower eGFR was observed in the single-dose ITT rATG group (multivariable *P* = 0.05). The coefficient of multiple determination, *R*^2^, for this seven-variable linear model was 0.48. While the prognostic factors identified via stepwise linear regression for eGFR at 12 and 24mo post-transplant were similar, no noticeable association between the single-dose ITT rATG group and eGFR at 24 mo was observed (multivariable *P* = 0.50; results not shown).

**Table 10:** Final multivariable linear regression model for eGFR at 12 mo post-transplant re-run with additional adjustment for the joint effects of single-dose ITT rATG induction and its propensity score (*N* = 90).

	Univariable	Multivariable model^a^	
Variable	*P*-value	*P*-value	Coeff ± SE
Developing a first BPAR by 12 mo	0.000009	0.00001	−29.062 ± 6.213
Taller recipient height at 12 mo (cm)	0.00004	0.0001	−0.381 ± 0.094
Male recipient	0.45	0.03	−9.687 ± 4.514
Longer CIT (h)	0.01	0.005	−0.599 ± 0.207
Older donor age (years)	0.23	0.03	−0.524 ± 0.242
Single-dose ITT rATG induction	0.02	0.05	−9.131 ± 4.573
Propensity score for single-dose ITT rATG induction	0.11	0.38	2.327 ± 2.660

a Variables that were included in this linear regression model were defined as follows: developing a first BPAR by 12 months post-transplant ={1 if a first BPAR occurred by 12 months post-transplant, 0 otherwise}, recipient height (cm) at 12 months post-transplant (continuous variable), male recipient (zero–one variable) {=1 if the recipient is male, 0 otherwise}, CIT (h) (continuous variable), donor age (years) (continuous variable), single-dose ITT rATG induction (zero–one variable) {=1 if single-dose ITT rATG induction, 0 otherwise}, and propensity score for single-dose ITT rATG induction [continuous variable, determined by the logistic regression model in Table 4 as −3.939 + 0.101*recipient age (years) + 0.097* warm ischemia time (min)]. The coefficient of multiple determination, *R*^2^, achieved for this seven-variable linear model was 0.48.

## Discussion

We believe that this retrospective study of 92 pediatric kidney-alone transplant recipients at a single center provides the following important clinical insights into pediatric kidney transplantation: (i) dual induction with rATG and basliximab, combined with a maintenance regimen including rTd, full IMPDH inhibitor dosing, and corticosteroid avoidance, is associated with favorable clinical outcomes at 12 mo post-transplant, including freedom from first BPAR, freedom from any viremia, and overall graft survival; (ii) the use of three rATG induction doses (compared with a single dose) may offer some benefit in terms of a reduced hazard of first BPAR (particularly, in achieving fewer borderline and grade IA rejections) and a somewhat more favorable eGFR at 12 mo post-transplant; simultaneously, there appears to be no independent detrimental effect of using three rATG induction doses on the hazard of developing any viral viremia. These results are consistent with previous reports in adult kidney transplantation evaluating dual induction with rATG and basiliximab (5–10).

Our results also suggest that the development of a first BPAR (and its treatment) has no notable effect on the subsequent risk of developing viral viremia in pediatric kidney transplant recipients. Similarly, the development of BK viremia does not appear to affect the subsequent risk of developing a first BPAR, whereas CMV viremia is associated with an increased risk of subsequently developing a first BPAR (23). These results therefore suggest that, among pediatric kidney transplant recipients, increasing immunosuppression to treat or prevent rejection may not lead to a concomitant increase in the risk of developing viral viremia.

As stated earlier in this report, basiliximab (anti-CD25) was added to rATG (thymoglobulin) after clinical observations suggested a limited anti-CD25 effect with rATG therapy alone, as well as to ensure immunosuppression of alloantigen-stimulated effector T cells expressing CD25 (5). Depletion of CD3+ cells with rATG—either alone or in combination with rATG/basiliximab—resulted in signiﬁcantly greater reductions in peripheral blood CD3+ cells compared with basiliximab alone. However, rATG alone did not sustain suppression of CD25 cells to the same extent as rATG/basiliximab. Effector T cells expressing CD4 and CD25 are responsible for allorecognition and alloresponsiveness (5) and have been identiﬁed in the peripheral blood of human transplant recipients (5). Thus, any additional reduction in CD25-bearing cells achieved with rATG/basiliximab therapy can be important, indicating a more targeted effect on effector T cells.

In this pediatric kidney transplant study, we found that both African-American (non-Hispanic) and Hispanic recipients had a much higher hazard rate of developing a first BPAR compared with white (and Asian) recipients. In other pediatric kidney transplant studies, significantly higher rates of BPAR and death-censored graft failure have been reported among African-American recipients (1, 24, 25), older recipients (1, 24, 26, 27), and those with greater HLA mismatches (24–27). Clearly, these findings suggest that higher rATG induction dosing may be warranted in these higher-risk patient subgroups. Conversely, we also showed that younger transplant recipients, particularly those <7 years of age at transplant, had significantly higher rates of BK viremia post-transplant. Thus, an argument can be made in favor of using lower rATG induction dosing in selected younger transplant recipients (without dramatically increasing their first BPAR rate).

Viral infections have recently been reported to be associated with poorer renal function and graft loss among pediatric kidney transplant recipients (28). While we found no such association betwen the development of any viral viremia and impaired renal function during the first 24 mo post-transplant, one patient in the single-dose ITT rATG group, who was 10 years of age at transplant, experienced graft failure due to a combination of CAI and chronically high parvovirus infection at 13.3 mo post-transplant. Thus, viral infections continue to be a cause of post-transplant morbidity.

Long-term corticosteroid therapy is still considered by many to be a standard component of maintenance immunosuppression in pediatric kidney transplant recipients; however, it is associated with multiple long-term side effects. A recent review found that corticosteroid withdrawal/avoidance in pediatric renal transplantation was associated with significant improvements in growth, particularly among prepubertal patients (29). In this study, we were able to maintain corticosteroid avoidance (i.e., defined as not requiring corticosteroids for ≥3 mo) in 62% (57/92) of our cohort during the first 24 mo post-transplant. Corticosteroid maintenance in pediatric kidney transplantation is also associated with a higher incidence of NODAT, which can lead to poorer renal function and an increased risk of graft loss (30). Our observed low incidence of NODAT (2 of 92 patients) during the first 24 mo post-transplant was probably attributable to our use of rTd and early corticosteroid withdrawal. None of our kidney allograft recipients suffered from a decline in renal function due to NODAT.

The main limitation of this study is that the two ITT rATG dose groups were compared in a retrospective non-randomized fashion (rather than in a prospective randomized trial), as well as the presence of limited statistical power to detect small-to-moderate differences (with only 42 patients in the three-dose ITT rATG group and 50 patients in the single-dose ITT rATG group). Despite our best efforts to carefully compare these two ITT groups, a non-negligible discrepancy remained (as highlighted in the Results section and Table 2) between planned and actual rATG exposure. In the single-dose group, 20.0% of patients received additional doses, while in the three-dose group, 19.0% patients did not complete the full regimen. This overlap in actual exposure may limit the interpretability of the comparisons made between the two ITT groups, as the analysis does not strictly represent a “single-dose vs. three-dose” contrast.

Furthermore, a more complete evaluation of the clinical benefits of dual-agent induction with rATG/basiliximab versus single-agent induction in pediatric kidney transplantation would require a prospective randomized trial. While most studies evaluating single-agent rATG induction in pediatric kidney transplantation have used higher dosing (typically, 1.5 mg/kg/day for 4–6 days intraoperatively and during the first post-transplant week) (31–36) than that used in our pediatric cohort, the distinct benefits and drawbacks of using higher vs. lower rATG dosing in this population remain unclear. One clear finding from this study is that the development of BPAR has a significantly negative effect on renal function, as shown by our multivariable eGFR results; therefore, reducing the risk of BPAR should remain a key priority when choosing an appropriate induction therapy strategy in pediatric kidney transplantation, even when considering potential drawbacks.

Limitations in drawing conclusions from a single-center study also apply, and generalization of our study results to other pediatric populations will require independent validation. In addition, the prognostic impact of withholding or discontinuing mycophenolate acid (due to leukopenia, gastrointestinal issues, or persistent viral viremia) was not analyzed, nor were tacrolimus trough levels at the time of rejection occurrence. Baseline donor and recipient CMV and EBV serostatus were also not retrieved for this study, which may have aided in interpreting post-transplant viral events. Despite these limitations, most of each patient's collected baseline and clinical outcome data were carefully retrieved and analyzed using state-of-the-art statistical methods, and a cohort of 92 pediatric kidney transplant recipients is substantial (by current standards). Importantly, comparing the safety and efficacy of using three vs. only one rATG induction dose using an ITT approach, even in a retrospective non-randomized study, remains important, as reasons for not receiving the planned induction dose could certainly influence patients' clinical outcomes (i.e., comparisons based on actual rATG dose received would introduce treatment selection bias).

In conclusion, our attempt to improve long-term graft and patient survival in pediatric kidney transplantation using innovative immunosuppression strategies—such as dual rATG/basiliximab induction, reduced tacrolimus dosing, and corticosteroid avoidance, which were designed to reduce BPAR rates without increasing the rates of other adverse events (such as viral viremias)—appears somewhat more promising with three rATG induction doses (vs. using only one induction dose). This attempt will require (planned) further follow-up to more clearly demonstrate the relationship between rATG induction dosing strategies and long-term clinical outcomes.

## Data Availability

The raw data supporting the conclusions of this article will be made available by the authors, without undue reservation.
